# Wheat germ *in vitro* translation to produce one of the most toxic sodium channel specific toxins

**DOI:** 10.1042/BSR20140050

**Published:** 2014-07-29

**Authors:** Wael Gad, Rahma Ben-Abderrazek, Khadija Wahni, Didier Vertommen, Serge Muyldermans, Balkiss Bouhaouala-Zahar, Joris Messens

**Affiliations:** *Brussels Center for Redox Biology, 1050 Brussels, Belgium; †Structural Biology Research Center, VIB, 1050 Brussels, Belgium; ‡Structural Biology Brussels Laboratory, Vrije Universiteit Brussel, 1050 Brussels, Belgium; §Laboratoire des Venins et Molécules Thérapeutiques, Institut Pasteur de Tunis-Université Tunis El Manar, Tunisia; ∥de Duve Institute, Université Catholique de Louvain, 1200 Brussels, Belgium; ¶Cellular and Molecular Immunology, Vrije Universiteit Brussel, 1050 Brussels, Belgium; **Medical School of Tunis, University of Tunis El Manar, El Manar, Tunisia

**Keywords:** AahII, anti-venom, dromedary, recombinant protein, scorpion toxin, single-domain antibody, wheat germ extract, Aah, *Androctonus australis hector*, NbAahII10, *Androctonus australis hector* nanobody 10, CBB, Coomassie Brilliant Blue, CDR1, complementary-determining region 1, GST, glutathione S-transferase, *h*QSOX1b, human quiescin-sulphhydryl oxidase, i.c.v., intracerebroventricular, IMAC, immobilized metal-ion-affinity chromatography, LD_50_, median lethal dose, PDI, protein disulphide isomerase, QSOX, Quiescin sulfhydryl oxidase, rAahII, recombinant AahII, TEV, tobacco etch virus, WGE, wheat germ embryo

## Abstract

Envenoming following scorpion sting is a common emergency in many parts of the world. During scorpion envenoming, highly toxic small polypeptides of the venom diffuse rapidly within the victim causing serious medical problems. The exploration of toxin structure-function relationship would benefit from the generation of soluble recombinant scorpion toxins in *Escherichia coli*. We developed an *in vitro* wheat germ translation system for the expression of the highly toxic Aah (*Androctonus australis hector*)II protein that requires the proper formation of four disulphide bonds. Soluble, recombinant N-terminal GST (glutathione S-transferase)-tagged AahII toxin is obtained in this *in vitro* translation system. After proteolytic removal of the GST-tag, purified rAahII (recombinant AahII) toxin, which contains two extra amino acids at its N terminal relative to the native AahII, is highly toxic after i.c.v. (intracerebroventricular) injection in Swiss mice. An LD_50_ (median lethal dose)-value of 10 ng (or 1.33 pmol), close to that of the native toxin (LD_50_ of 3 ng) indicates that the wheat germ *in vitro* translation system produces properly folded and biological active rAahII. In addition, NbAahII10 (*Androctonus australis hector* nanobody 10), a camel single domain antibody fragment, raised against the native AahII toxin, recognizes its cognate conformational epitope on the recombinant toxin and neutralizes the toxicity of purified rAahII upon injection in mice.

## Abstract 2

A wheat germ embryo derived cell-free translation system expresses a biologically active, highly toxic scorpion venom protein that is fully neutralized by a camel single domain antibody fragment raised against the native scorpion toxin.

## INTRODUCTION

**Figure 1:**
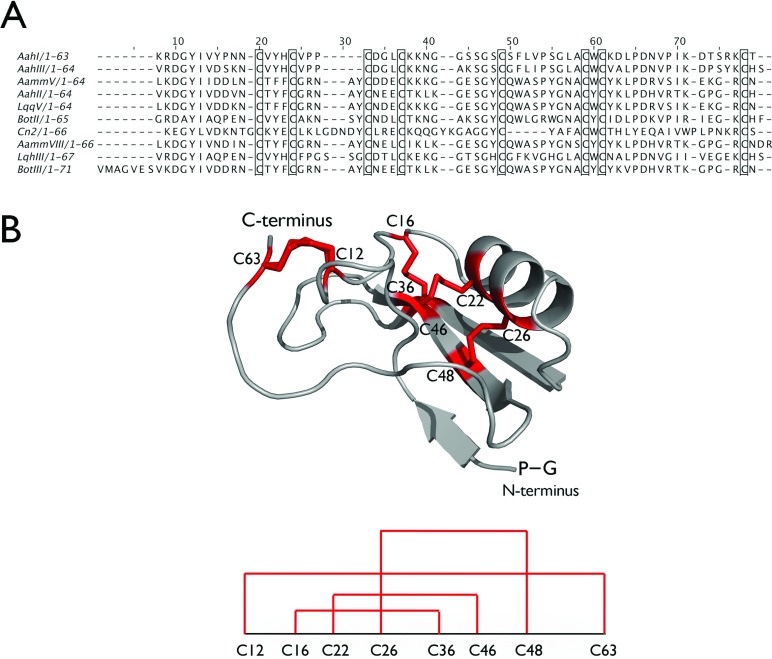
The amino acid sequence and structure of scorpion toxin (**A**) ClustalW alignment [44] of the amino acid sequences of representative scorpion toxins is shown. *Androctonus australis hector* AahI (P01479), *Androctonus australis hector* AahIII (P01480), *A. mauretanicus mauretanicus* Amm V (P01482), *Androctonus australis hector* AahII (P01484), *Leiurus quinquestriatus quinquestriatus* LqqV (P01481), *Buthus occitanus tunetanus* BotII (P01483), *Centruroides noxius* Cn2 (AAB21461) (β-toxin), *A. mauretanicus mauretanicus* Amm VIII (Q7YXD3), *L. q. hebraeus* LqhIII (P56678) (α-like toxin), *Buthus occitanus* BotIII (P01485). The conserved cysteines are boxed. (**B**) The ribbon presentation of the structure of rAahII toxin (PDB 1PTX) [14,45]. The toxin contains four disulphide bonds between non-consecutive cysteines, according to the scheme depicted below. The amino acid sequence of the rAahII toxin after removal of the GST-tag contains two extra N-terminal residues (glycine and proline) that are indicated.

The many accidents involving scorpion stings represent a real medical emergency in the Middle East and the south Mediterranean regions [1]. Aah (*Androctonus australis hector*) is one of the most toxic scorpions to humans and is often implicated in fatal scorpion sting accidents in Algeria and Tunisia [2]. The toxicity of the Aah scorpion venom is essentially due to the presence of three small basic toxins (7 kDa) that act on the voltage-gated sodium channels of excitable cells [3]. The family of toxins displays common cysteine-stabilized α-helix and a three-stranded β-sheet motifs in a βαββ organization [4]. Because of sequence variability, a broad antigenic polymorphism is displayed in this family of proteins [5]. Aah toxins belong to one out of two distinct structural and antigenically unrelated groups: group I (polypeptides AahI, AahIII and AahIV) and group II (AahII) [3,5,6]. Amino acid sequence alignment showed that the cysteins are highly conserved among toxins of this family (Figure 1A). They play a crucial role in the functional architecture and structural stability of the toxins.

AahII toxin is the most poisonous toxin among all North African scorpions with an LD_50_(median lethal dose)<3 ng upon i.c.v (intracerebroventicular) administration in Swiss mouse of ~20 g [7]. AahII has been purified from scorpion venom [8,9] and its structural and antigenic properties are well established [4,10]. It displays the highest affinity for site 3 of the neuronal Na_v_1.2 and muscular Na_v_1.4 channels in mammals [11]. The functional surface of LqhII, the toxin of *Leiurus quinquestriatus hebreaus* that differs remarkably only in its N- and C-termini with AahII, has been determined, as well as the docking of this protein at the voltage-dependent sodium channel [12,13]. Immunochemical analysis of AahII toxin had led to the identification of four antigenic regions, nearby the α-helix, in the N- and C-terminal regions, and in a surface loop specific to α-toxins [10,14,15]. Immunotherapy remains probably the most efficient treatment after envenomation, but the outcome depends on both accurate identification of the scorpion species involved and the timely anti-venom administration [16]. Because of their high affinity and specificity, small size and robust behaviour, the single-domain antibodies, referred to as Nbs (nanobodies), have been proposed to substitute the polyclonal Fab’_2_ to treat the scorpion envenoming [17,18]. Indeed, a bispecific Nb construct comprising an Nb neutralizing AahI’ toxin and an Nb neutralizing AahII toxin was proven to protect mice and rats that received a subcutaneous lethal dose of the scorpion venom [3].

The progress in the molecular dissection of scorpion α-toxins and their structure and function is slow due to the difficulty of producing soluble recombinant bioactive toxins [19]. For structural and functional studies on toxins, an efficient *Escherichia coli* expression system that results in unlimited amounts of soluble, properly folded toxin is desired. Early attempts to produce rAahII (recombinant AahII) in microorganisms yielded only minor amounts of largely insoluble material that required tedious *in vitro* refolding steps [16,20,21]. However recently, the LqhhII toxin and BMαTX14 toxins were refolded from inclusion bodies in single digit mg amounts per litre bacterial culture into soluble recombinant toxins showing similar biological activities as those of the native proteins [19,22]. Despite these recent successes, apparently it remains a major challenge to express large amounts of soluble toxin without *in vitro* refolding. Although it looks a promising strategy, it remains challenging to refold a 64 amino acid long peptide and oxidize eight cysteines in four correct disulphide bridges and to display the right surface epitopes necessary for its full toxicity and antigenicity. Structural and antigenic characterizations showed that recombinant scorpion toxins possess a structural flexibility that leads to the accommodation of enforced modifications in the final protein fold [23].

Here, we evaluate an eukaryotic cell-free translation system based on the WGE (wheat germ embryo) [24] for the expression of a highly toxic AahII scorpion venom protein. As this rAahII protein (7.4 kDa) has to form four disulphide bonds between cysteines located in non-consecutive positions (Figure 1), it is an excellent model for studying the capacity of the WGE system for the expression of proteins bearing multi disulphides. Furthermore, it had been described that the WGE expression system contains the cellular machinery to form complex conformational disulphide bonds [25]. We also studied the role of the oxidoreductases, *h*QSOX1b (human quiescin-sulphhydryl oxidase) and *h*PDI (human protein disulphide isomerase), as possible protein-folding catalysts. Finally, we investigated the toxicity of this rAahII (after removal of the GST (glutathione S-transferase) tag which leaves two extra amino acids at the N-terminal of the rAahII relative to the native AahII) and the potential of nanobody NbAahII10 (*Androctonus australis hector* nanobody 10), raised against native AahII toxin [3,17,26], to neutralize the toxicity of the purified rAahII toxin. Our results demonstrate the successful expression of soluble and active rAahII toxin and the ability of NbAahII10 to neutralize its toxicity in mice.

## EXPERIMENTAL

### AahII toxin, *h*QSOX1b and *h*PDI cloning into pEU vector

The gene for AahII scorpion toxin (GeneBank accession number P01484) was synthesized by Integrated DNA Technologies (Belgium). The coding sequence (Met1**–**His65) was ligated into the pEU-vector (CellFree Sciences) without any tag or with an N-terminal GST-tag [with both a 3C protease (two extra N-terminal amino acids after cleavage) and a TEV (tobacco etch virus) protease (18 extra amino acids after cleavage) cleavage site] or His-tag. This plasmid vector is specially designed for the wheat-germ cell-free expression system [24] in combination with the SP6 RNA polymerase transcription system. Briefly, the coding sequence of the scorpion toxin gene was amplified by PCR and introduced into pEU vector using XhoI and SmaI restriction and EcoRV and BamHI sites, respectively.

The *h*QSOX1b (R32-I604, GeneBank accession number NP_001004128.1) without signal peptide and *h*PDI (A18-L508, GeneBank accession number NP_000909.2) without signal peptide gene fragments were cloned with a FLAG-tag and GST tag at the N-terminal position into the pEU-GST-MCS vector. The coding sequence of *h*QSOX1b and *h*PDI were amplified by PCR and introduced into the BamHI and SmaI and the XhoI and SmaI restriction sites, respectively. All constructs were confirmed by sequencing analysis performed at the VIB genetic facility (GSF).

### AahII toxin cloning and expression in *E. coli*

The AahII scorpion toxin gene was amplified by PCR from the pEU-AahII-GST construct and introduced into pET28a vector between BamHI and HindIII sites.

Plasmid DNA of pET28-GST-AahII transformed into *E. coli* SHuffle™ T7 Express (BioLabs) and Origami™ DE3 competent cells (Novagen). Transformed bacteria were grown at 37°C in the LB [Luria–Bertani (broth)] medium supplemented with 25 μg/ml Kanamycin, until *A*_600 nm_ of 0.6. At this cell density, the expression was induced with 1 mM IPTG (isopropyl-b-D-thiogalactopyranoside), and cultured overnight at 30°C. Harvested cells were resuspended in 50 mM potassium phosphate, pH 7.5, 300 mM NaCl, 0.1 mg/ml lysozyme, 0.1 mg/ml AEBSF [4-(2-aminoethyl)benzenesulfonyl fluoride] and 0.1 μg/ml leupeptine. Cells were broken at 4°C by three sonications for 1 min each and centrifuged for 30 min at 14000 rpm. For identification, protein fractions of supernatant (7.5 μl) and pellet (7.5 μl) were analysed by non-reducing SDS/15%PAGE and Western blot detection using anti-GST antibody (EnoGene).

### Small-scale transcription and translation reaction

Plasmid DNA of the pEU-AahII scorpion toxin (+GST, +His-tag and without tag) was extracted using the Maxiprep DNA extraction kit (Invitrogen) and extra purified using the phenol/chloroform method to remove all RNase contamination [24]. DNA (2 μg) of each construct was transcribed using SP6 RNA polymerase, 25 mM NTP mix, RNase inhibitor and 5× transcription buffer (CellFree Sciences) for 6 h at 37°C without shaking. The mRNA was cooled to avoid degradation and checked on a 1% (w/v) agarose gel. For translation, the mRNA of each construct (10 μl) mixed with the same amount of the WGE WEPRO 7240G (CellFree Sciences) and 0.1 mg of creatine kinase to make the bottom layer, was incubated with the 1× SUB-A MIX SGC (upper layer), which includes the amino acid mixture (CellFree Sciences). This was incubated at 15°C for 20 h without shaking in a 96-well plate (Greiner bio-one) using a small Eppendorf tube Thermomixer (Roche).

### Co-expression of rAahII toxin with *h*QSOX1b and/or *h*PDI

pEU FLAG-tag *h*QSOX1b, FLAG-tag *h*PDI and GST-tag, His-tag and no tag rAahII toxin (2 μg each) were transcribed separately using SP6 RNA polymerase, 25 mM NTP mix, RNase inhibitor and 5× transcription buffer. The mRNA of respectively rAahII toxin, *h*QSOX1b and *h*PDI were translated at 26°C for 10 min using the WGE WEPRO7240G. The mRNA of rAahII toxin (10 μl) was then mixed with the same amount of mRNA from *h*QSOX1b, *h*PDI and *h*QSOX1b+*h*PDI to make the bottom layer and incubated at 15°C for 20 h without shaking in a 96-well plate with 206 μl of the SUB-A MIX SGC (upper layer) which includes the amino acid mixture for each reaction. After the incubation, the expressed proteins were evaluated on a CBB (Coomassie Brilliant Blue)-stained 15% non-reducing SDS/PAGE, and by Western blot using anti-His (Sigma Aldrich), anti-GST antibody (EnoGene) and NbAahII10.

### NbAahII10 C/S expression and purification

Identification of NbAhII10 was described previously [26]. The amino acid sequence analysis showed that NbAahII10 contains a single cysteine in its CDR1 (complementary-determining region 1) [17], which leads to dimerization of the Nb upon storage. To avoid this dimerization, the cysteine was substituted by serine and the resulting NbAahII10 C/S was shown to be identical to freshly prepared NbAahII10 in recognizing its cognate antigen.

For large-scale production of the recombinant His-tagged NbAahII10 C/S, 6 litres of Terrific Broth supplemented with 0.1% (w/v) glucose and 100 μg/ml ampicillin was grown at 37°C until an *A*_600nm_ between 0.6 and 0.9 was reached. Expression of the recombinant protein was induced with 1 mM isopropyl β-D-thiogalactoside (Sigma Aldrich) and the culture was further incubated at 28°C for 16 h. After pelleting the cells, the periplasmic proteins were extracted by osmotic shock [17,18], and loaded onto Ni^2+^-Sepharose column (GE Healthcare) for IMAC (immobilized metal-ion-affinity chromatography) equilibrated in 50 mM sodium phosphate buffer solution (pH 7.0), 1 M NaCl. The unbound proteins were washed out with ten column volumes of 50 mM sodium phosphate buffer solution (pH 7.0), 1 M NaCl, followed with 30 column volumes of 50 mM Na phosphate buffer solution, 1 M NaCl (pH 6.0). The recombinant protein was eluted from the resin with 5 ml of 50 mM Na acetate, 1 M NaCl (pH 4.7). The pH of the fractions under the elution peak was increased by adding 1 M Tris–HCl (pH 7.5). The fractions under the elution peak were concentrated on a 3.5 kDa molecular mass cut-off filters (Millipore Belgium) and loaded onto a Superdex75 10/30 gel-filtration column (GE Healthcare) equilibrated with PBS (pH 7.5). The purity of the protein fraction was evaluated by 15% non-reducing SDS/PAGE and stained with CBB. The absorption at 280 nm and the theoretical extinction coefficient were used to determine the protein concentration [27].

### Immunodetection using ELISA, dot blot and Western blot

The expressed rAahII toxin proteins have been evaluated by SDS/15%PAGE stained with CBB or by Western blot using polyclonal AahII-specific rabbit antibody, anti-GST antibody (EnoGene), anti-His antibody (Sigma Aldrich) or AahII toxin-specific NbAahII10 C/S. The ELISA, Western blot and dot blot assays were used to assess the ability of the purified rAahII toxin to be recognized by different antibodies. Briefly for ELISA, samples of 1 μg/ml purified GST-rAahII (cleaved and non-cleaved) were coated on maxisorb plates (Nunc). After blocking of residual protein-binding sites with PBS, 5% non-fat dried skimmed milk powder, polyclonal AahII-specific rabbit antibodies (1/5000 dilution), anti-GST (1/10000), anti-His (1/10000) or AahII-specific NbAahII10 antibody 5 μg/ml [17,26], were incubated, 1 h at 37°C. Immunoreactivity was revealed using a goat anti-rabbit IgG peroxidase conjugate (1/1000), anti-rabbit IgG alkaline phosphatase (1/1500) or an anti-mouse IgG alkaline phosphatase (1/1500), respectively. Absorbance was measured at 492 nm, after 15 min incubation with the conjugate–enzyme substrate at room temperature. ELISA assays were repeated twice.

### Up-scaling of the GST–rAahII toxin fusion with and without *h*QSOX1b

For up-scaling, plasmid DNA of GST–rAahII toxin (25 μg) and FLAG-tag *h*QSOX1b (25 μg) were transcribed separately for 6 h at 37°C using SP6 RNA polymerase, 25 mM NTP mix, RNase inhibitor and 5× transcription buffer (CellFree Sciences). The mRNA was cooled to avoid degradation and checked on 1% (w/v) agarose gel. For translation the mRNA (250 μl) was mixed with the same amount (250 μl) of the WGE WEPRO 7240G and 1 mg of creatine kinase to make the bottom layer. The 5.5 ml of 1X SUB-A MIX including an amino acid mixture (CellFree Sciences) was used for translation at 15°C for 20 h without shaking in a six-well plate in Thermomixer incubator (Roche). The expressed protein was evaluated, before purification, on SDS/15%PAGE stained with CBB and by Western blot using anti-GST antibody (EnoGene) and scorpion toxin-specific NbAahII10. For co-expression, mRNA from GST–rAahII toxin and FLAG–*h*QSOX1b were translated separately for 10 min at 26°C using WGE WEPROG. After 10 min incubation, mRNA of GST–rAahII toxin was mixed with the same amount of mRNA from *h*QSOX1b and translation was continued at 15°C for 20 h without shaking.

### Purification of GST–rAahII fusion and cleavage with rhinovirus 3C protease

For purification, two batches (6 ml each) of GST–rAahII fusion (expressed with and without *h*QSOX1b) were spun down at 14000 rpm for 20 min at 4°C. The supernatant (6 ml) was bound to 0.5 ml Glutathione Sepharose™ beads (GE Healthcare) equilibrated with buffer 50 mM Tris/HCl pH 7.5, 150 mM NaCl, for 2 h at 4°C. The mixture loaded on a PD10 column, and washed with three-column volumes of the same buffer solution and the GST fusion was eluted with (2 ml) 50 mM Tris/HCl, pH 7.5, 10 mM reduced L-glutathione. Eluted proteins were dialysed for 4 h at 4°C against PBS (pH 7.5) with two buffer changes. The purity of the purified protein was evaluated by non-reducing SDS/15%PAGE and by a Western blot using anti-GST antibody. The GST-tag was cleaved overnight at 16°C using human rhinovirus 3C protease enzyme (prepared in-house). A 10-kDa Millipore concentrator was used to separate the uncleaved GST-tagged rAahII (33 kDa) from cleaved rAahII (7 kDa). The 2 ml flow through was concentrated on a 3.5-kDa concentrator to 20 μl, and the protein concentration was determined based on the theoretical extinction coefficient at 280 nm for the rAahII amino acid sequence, which gives an absorbance for a 0.1% solution of the rAahII toxin of 2.26. The endotoxin level of the purified rAahII protein was measured using LAL (*Limulus* amoebocyte lysate) assay. The same values were obtained for the rAahII protein extract as for PBS (negative control)(results not shown).

### MS analysis

Intact protein mass measurements were performed by direct infusion in a micro electrospray ionization ion trap mass spectrometer (LTQ XL, ThermoFisher Scientific). Briefly, purified TEV protease cleaved rAahII toxin was desalted using C18 Spin Columns (Thermo Scientific) and eluted in 50% (v/v) acetonitrile 0.1% (v/v) acetic acid. The mass spectrometer was operated manually in positive ion mode with a source voltage set at 3.8 kV and the ion transfer tube at 220°C. The parent ions were submitted to SID (in-source dissociation) using the minimal energy to promote efficient declustering of water molecules and salts adducts. The mass spectra were deconvoluted using the software ProMass Deconvolution from ThermoFisher Scientific.

### Toxicity assay and LD_50_ determination

All experiments on mice were carried out in accordance with the European Community Council Directive (86/609/EEC) for experimental animal care, and all procedures met with the approval of the Institutional Research Board of the Pasteur Institute of Tunis. A cohort of four to six healthy male Swiss mice (20±2 g) was used to estimate the toxicity of the native AahII or purified recombinant 3C protease cleaved rAahII toxins. Samples of 5 μl native AahII (3 ng), increasing amounts of freshly prepared rAahII toxin (4, 5, 6, 8, 10, 15 and 20 ng) and increasing amounts of freeze–thawed rAahII toxin (4, 6, 8, and 10 ng) in PBS-containing buffer were individually injected by i.c.v. route. As negative control, mice received only PBS buffer or a 10 ng unrelated protein (*Urtica dioica* agglutinin protein [28]), which has been produced following exactly the same procedure as described for the rAahII toxin. As positive control, native AahII toxin purified as described previously [26] was used. Deaths within each cohort of four mice were monitored 24 h after injection. The LD_50_ was determined as the amount of toxin whereby 50% of the mice survived the injection [17,29]. All assays were repeated three times to assess the reproducibility.

### Neutralization assay

For the *in vivo* neutralization assays, we mixed an increasing lethal dose of purified 3C protease cleaved rAahII toxin with a ~2-fold molar excess of NbAahII10 C/S in a total volume of 5 μl and incubated the mixture for 1 h at 37°C before i.c.v. injection [26]. In all cases, the mouse survivals were monitored 24 h after injection. Native AahII toxin was used as a positive control. Negative control mice received only PBS buffer or the unrelated *Urtica dioica* agglutinin protein. The NC (neutralization capacity) was calculated according to the guidelines proposed by the WHO (1981): [NC (LD_50_/mg of Nb)=(LD_50_ in presence of Nb−LD_50_ in absence of Nb)/mg of Nb].

## RESULTS AND DISCUSSION

### Wheat germ *in vitro* translation system expressing soluble GST-tagged rAahII toxin

Previous studies showed that the expression of recombinant scorpion toxins in *E. coli* [19,30–35] and in yeast [36] produced insoluble toxins that could be refolded to yield 1–4 μg/l culture. In addition, the yield obtained with monkey kidney COS-7 cells was extremely low (0.2 μg/10^6^ cells) [20]. Nevertheless the pET-14 expression system seemed to produce large quantities of inclusion bodies in bacteria, which could be solubilized and *in vitro* refolded into mg amounts per litre of culture. The aggregation of recombinant toxin might be due to the presence of large solvent exposed hydrophobic patches (e.g. the AahII-binding site onto the sodium channel site 3 relies on a large hydrophobic surface [12]); however, the absence of disulphide bond formation between the eight cysteines of the scorpion toxin in the cytoplasm is possibly the major factor for the insolubility. We therefore decided first to target the expression of rAahII toxin with an N-terminal GST-tag to the cytoplasm of SHuffle™ T7 Express cells. In SHuffle™ T7 Express cells, the genes for glutaredoxin reductase and thioredoxin reductase have been deleted, which allows disulphide bonds to form in the cytoplasm [37–39]. Furthermore, SHuffle cells express a version of the periplasmic disulphide bond isomerase DsbC that lacks its signal sequence, retaining it in the cytoplasm. The DsbC enzyme has been shown to act on proteins with multiple disulphide bonds, to correct mis-oxidized bonds and promote proper folding [40]. At the same time, we expressed GST–rAahII toxin using Origami™ DE3 cells. Proteins in the soluble and insoluble fractions were evaluated on non-reducing SDS/15%PAGE gels stained with CBB, or by Western blot using anti-GST antibody (results not shown). All these conditions failed to reveal a band corresponding to the size of GST–rAahII toxin. This indicates that even these modified *E. coli* based systems are not suitable for the expression of soluble GST–rAahII toxin.

**Figure 2:**
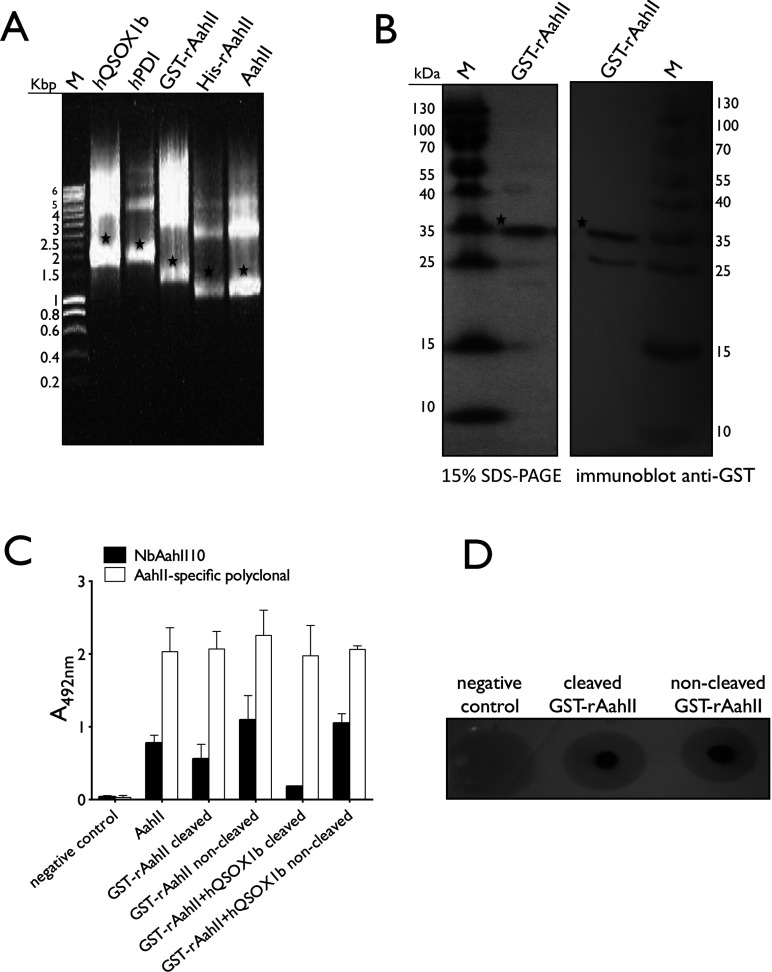
Expression of GST–rAahII toxin fusion with WGE and immuno-characterization (**A**) Ethidium bromide-stained agarose gel showing the mRNA of *h*QSOX1b, hPDI, GST–rAahII, His–rAahII and rAahII toxin without tag after transcription. Stars indicate the specific mRNA bands. (**B**) Non-reducing SDS/15%PAGE showing the migration of the purified GST–rAahII fusion (33 kDa) next to a pre-stained size marker (Fermentas) (left). The Western blot (right) is developed using an anti-GST antibody (EnoGene). Stars indicate the protein bands of the GST–rAahII fusion. (**C**) Results of an ELISA with cleaved and non-cleaved purified GST–rAahII toxin (with and without oxidoreductase *h*QSOX1b) (~300 ng/well) as revealed with a rabbit polyclonal AahII-specific antibody and NbAahII10. As a negative control a non-related protein was used, as a positive control native AahII (100 ng/well). (**D**) Both cleaved and non-cleaved GST–rAahII toxin are recognized by NbAahII10 in dot blot experiment.

Thus, we decided to express rAahII toxin in the eukaryotic cell-free translation system based on the WGE [24]. The WGE comprises all components for translation, stored in a dried state, and ready for protein synthesis as soon as germination starts. Hence, the WGE extract complemented with mRNA has been documented to synthesize toxic proteins and eukaryotic multi-domain proteins in a folded state [25]. As such, we cloned the synthetic rAahII toxin gene into the pEU–vector with an N-terminal GST-tag, His-tag and without tag. This pEU plasmid vector had specifically been designed for the wheat-germ cell-free expression system [24] to operate in combination with the SP6 RNA polymerase transcription system. After 6 h of transcription at 37°C, the mRNA was cooled to avoid degradation and it was evaluated on a 1% (w/v) agarose gel (Figure 2A). The WGE translation mixture was incubated for 20 h at 15°C, and the expression of several rAahII toxin constructs was evaluated by non-reducing SDS/15%PAGE, by Western blot, and with an ELISA using anti-GST antibody, anti-His antibody, rabbit polyclonal AahII-specific antibody and NbAahII10 [17,26]. Apparently, the His-tagged toxin and the non-tagged toxin are not expressed, but the GST-tagged rAahII toxin was observed in the soluble fraction and is recognized with the anti-GST antibody in Western blot (Figure 2B), with the rabbit polyclonal AahII-specific antibody in ELISA (Figure 2C), and with NbAahII10 in ELISA and dot blot (Figure 2D). The soluble expression of the GST–rAahIII was independent of the cleavage site linker within the GST-construct.

### Co-expression with *h*QSOX1b or *h*PDI does not increase the yield of GST–rAahII toxin

Previously, we observed that the expression of disulphide bond rich proteins benefit from the presence of oxidoreductases, like *h*QSOX1b [41]. Properly folded scorpion toxin rAahII has to form four disulphide bonds (Figure 1B). Therefore we tested whether the expression of scorpion toxin might benefit from the presence of these oxidoreductases for the correct formation of disulphide bonds. We co-expressed the rAahII toxin in the wheat germ system with *h*QSOX1b and *h*PDI, respectively. The plasmids (2 μg each) encoding FLAG-tag *h*QSOX1b, FLAG-tag *h*PDI and GST-tag, His-tag and non-tagged rAahII toxin were transcribed separately with SP6 RNA polymerase. The mRNA was cooled and checked on a 1% agarose gel prior to translation (Figure 2A). The mRNA of rAahII toxin, *h*QSOX1b and *h*PDI, respectively, were translated for 10 min at 26°C using the WGE WEPRO7240G. For each reaction, the mRNA of rAahII toxin (10 μl) was mixed with the same amount of mRNA from *h*QSOX1b, *h*PDI and *h*QSOX1b+*h*PDI and incubated in a 96-well plate at 15°C for 20 h without shaking with 206 μl of the SUB-A mixture (upper layer). After the incubation, the expressed proteins were visualized on a CBB-stained non-reducing SDS/15%PAGE gel, and by Western blot developed with anti-His, with anti-GST antibody (Figure 2B), and an ELISA and dot blot with NbAahII10 (Figures 2C and 2D). Only the GST-rAahII toxin construct was expressed in soluble form, and co-expression with *h*QSOX1b or *h*PDI did not increase the final yield. Our results suggest that the presence of the GST-tag is beneficial for the folding of rAahII toxin in a wheat germ cell-free system, whereas no extra effect from disulphide forming oxidoreductases could be demonstrated.

### The rAahII toxin is recognized by a Nb raised against native toxin

**Figure 3:**
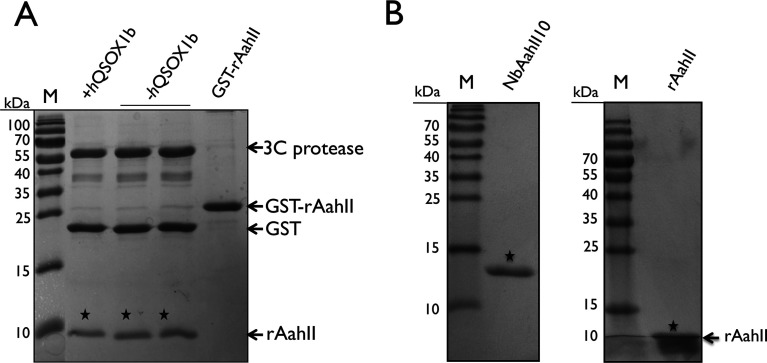
Non-reducing SDS–PAGE of rAahII (**A**)The migration of proteins on a non-reducing SDS/15%PAGE is shown for the purified non-cleaved GST–rAahII toxin (34 kDa, right-hand lane), and purified GST–rAahII toxin fusion expressed with and without *h*QSOX1b after cleavage with 3C protease. The rAahII toxin, GST and 3C protease enzyme migrate as a band of 7, 26 and 66 kDa, respectively. Stars denote the migration of the rAahII toxin bands. (**B**) A non-reducing SDS/15%PAGE showing the purified NbAahII10 C/S and rAahII toxin, both indicated by a black star.

In the next step, we purified GST–rAahII toxin in the presence and absence of *h*QSOX1b using Glutathione Sepharose™ beads. Target fusion proteins were eluted in 2 ml elution buffer and the GST-tag was cleaved off using human rhinovirus 3C protease (at 16°C for 12 h). Cleaved and non-cleaved proteins, expressed with and without oxidoreductase *hQSOX1b*, were evaluated by non-reduced SDS/15%PAGE (Figure 3A). Non-cleaved GST-toxin (34294 Da) migrates as a ~34 kDa protein indicating the presence of both GST (25750.9 Da) and toxin (7406.3 Da). Both antibodies, raised against AahII toxin and against GST, recognized purified GST–rAahII toxin (Figures 2B and 2C). The cleaved GST-toxin fusion proteins migrate after SDS/15%PAGE as separate bands of ~26 kDa (GST) and ~9 kDa (toxin+extra amino acids). Mass spectrometrical analysis after a tryptic digest of both bands confirmed their identity as GST and rAahII toxin, respectively.

In the next step, we wanted to test whether NbAahII10, a single domain antibody fragment raised against native AahII toxin [17,26], recognizes the cleaved recombinant toxin. Note that the cleaved rAahII protein contains two extra amino acids at its N-terminal end (Figure 1B). We expressed Nanobody NbAahII10 C/S in *E. coli* and purified it as described [17]. The IMAC purified protein elutes from the size exclusion chromatographic column as a single peak at the volume expected for a monomeric Nb and migrates after SDS/PAGE as a single band (Figure 3B). NbAahII10 C/S recognizes GST–rAahII toxin before and after cleavage with rhinovirus C3 in an ELISA and in a dot blot experiment (Figures 2C and 2D). However, after Western blot, NbAahII10 C/S failed apparently to recognize the toxin, which suggests that this Nb binds only to a conformational epitope, and not to a linear epitope. Since NbAahII10 C/S associates only with a conformational epitope of its cognate antigen, and since the native scorpion toxin is equally well recognized as the recombinant toxin, we conclude that at least this epitope of rAahII is correctly folded.

The automated deconvolution of the mass spectrometry peaks of the TEV-protease cleaved GST–rAahII construct resulted in an average mass of 9177.4 Da, which is very close to the calculated mass of 9176.2 Da for the recombinant protein with its four disulphide bonds formed. Next, we have tried to decipher the disulphide-bonding pattern using DBond software [42]. The AahII toxin has eight cysteines forming four non-consecutive disulphide bonds: C_12_–C_63_, C_16_–C_36_, C_22_–C_46_ and C_26_–C_48_. Unfortunately, it turned-out that the DBond software was not able to handle the identification of complex mixtures of disulphide-linked peptides as produced after tryptic digest of rAahII.

### Purified rAahII is toxic for mice

The toxicity of the recombinantly produced scorpion toxin was evaluated by an i.c.v. injection of purified rAahII into Swiss mice (20 g). Previously, this standard experimental approach has been shown to be effective in testing several toxins in a reproducible manner consuming a minimal amount of material [2,3,26]. The native toxin, purified from scorpion venom extract by HPLC [26], was i.c.v. injected as a positive control.

The systemic symptoms (irritability, agitation, mastication and tachycardia) were observed in mice that received a toxic dose of the native or rAahII. These symptoms were independent of the source of AahII toxin but varied in intensity, in time of occurrence and duration depending on the injected dose. The average number of survivors at 24 h after administration was assessed from two independent experiments, and the LD_50_-value was expressed as the amount of purified material (ng) injected into a mouse whereby 50% of the injected mice survived.

The LD_50_ of the native AahII injected i.c.v. in Swiss male mice (20 g in weight) is determined at 3 ng/mouse or 0.41 pmol/mouse (Table 1). With an LD_50_ value of 10 ng/mouse our freshly purified rAahII approached this value closely. In contrast, the *Urtica dioica* agglutinin protein, used as an unrelated protein, and a freeze-thawed de-activated sample of rAahII were not toxic at all in mice. These controls indicate that the purified rAahII toxin is properly folded and that the toxicity is caused by rAahII and not by the compounds of the solution.

**Table 1:** Toxicity of native AahII and purified rAahII after i.c.v. injection in Swiss mice Cohorts of four or six mice received various amounts of recombinant rAahII toxin (in a fixed volume of 5 μl) as indicated and surviving mice were counted 24 h later. *Urtica dioica* Agglutinin protein and 0.1% (w/v) BSA in 0.15 M NaCl were used as negative controls. The LD_50_ of native AahII was determined at 3ng/mouse [7] and that of rAahII at 10 ng/mouse.

Protein	Quantity injected (ng/mouse)	Surviving mice/total number of mice injected
Native AahII	3	2/4
Recombinant AahII	4	4/4
	5	4/4
	6	4/4
	8	5/6
	10	3/6
	15	0/6
	20	0/6
Freeze–thawed rAahII	4	4/4
	6	4/4
	8	4/4
	10	4/4
*Urtica dioica* agglutinin protein	10	4/4
0.1% BSA–NaCl	5 (μl)	4/4

To the best of our knowledge, this is the first time that a soluble rAahII toxin fraction has been found so toxic. Indeed, the 3-fold lower toxicity of the recombinant protein compared to the native AahII is far better than the previously reported hybrid construct of rAahII with maltose binding protein, which gave a 1000-fold lower toxicity (LD_50_ of 3 μg or ~62 pmol) after i.c.v. injection in mice [21].

The 3-fold difference in toxicity (3 ng for native AahII and 10 ng for rAahII) can be explained in two ways. It might be that only ~30% of the rAahII is folded into an active conformation, whereas ~70% of the protein is soluble although not folded into a toxic conformation. Alternatively, it might be argued that the reduced toxicity is attributed to the two additional residues at the N-terminal part of rAahII, and/or to the absence of C-terminal amidation of rAahII toxin expressed in the WGE eukaryotic cell-free system.

### NbAahII10 C/S neutralizes the toxic effect of the rAahII toxin

Since NbAahII10 C/S raised against native AahII recognizes recombinant toxin, we wanted to assess the neutralization capacity of this Nb on purified rAahII. Therefore we first incubated rAahII toxin with a 2-fold molar excess of NbAahII10 C/S and tested by i.c.v. injection in 20 g Swiss mice the toxicity of an increasing amount of this mixture corresponding to 1-, 1.5- or 2-fold the LD_50_ of rAahII. All mice survived this treatment (Table 2), proving that NbAahII10 C/S neutralizes the highly toxic dose of the recombinant rAahII toxin. Subsequently, we tested an equimolar ratio of the Nb to rAahII (1:1) and showed that 40 ng of NbAahII is effectively neutralizing 2 LD_50_ of the rAahII toxin in at least 66% of injected mice. Interestingly, ~50% of mice survived a 2 LD_50_ dose of i.c.v. injected rAahII when mixed with Nb at a 1/0.5 rAahII/NbAahII10 molar ratio. If only ~30% of the material were folded in an active, toxic conformation (remaining 70% folded in a soluble non-toxic conformation not recognized by the Nb), we calculated that 20 ng of the 2 LD_50_ would contain only 6 ng toxic rAahII protein that was recognized by NbAahII10 C/S. The presence of a nearly 2-fold molar excess of the Nb over the active rAahII is expected to fully neutralize all toxicity of rAahII. Since 4/6 or 3/6 mice survived the treatment, it rather indicates that the differences at the N- or C-terminal end between the native and rAahII are most likely causing the observed 3-fold reduced toxicity. These data are in line with the studies of Alami et al. [11]. They showed that the AmmVIII toxin of the *Androctonus mauretanicus mauretanicus* scorpion venom, which is 87% analogues to AahII but with a striking different C-terminal end, is totally devoid of toxicity. Hence the absence of toxicity is attributed to an additional C-terminal Asp, which is not present in the AahII toxin. This extra C-terminal residue has been suggested to induce steric hindrance with the receptor site, which was also demonstrated for K^+^ channel blockers [43].

**Table 2:** Neutralization of purified rAahII toxin by NbAahII10 C/S The *in vivo* neutralization was assessed after pre-incubating a molar excess (as indicated) of the NbAahII10 C/S with the rAahII toxin for 90 min at 37°C, before i.c.v. injection in Swiss mice (cohorts of six mice). Surviving mice were counted 24 h later.

	NbAahII10		
Quantity ng/mice rAahII	Molar ratio rAahII/NbAahII10	Quantity (ng/mice)	Surviving mice/total number of mice injected
10 (~1 LD_50_)	1:2	40	6/6
15 (~1.5 LD_50_)	1:2	60	6/6
15 (~1.5 LD_50_)	1:1	30	6/6
20 (~2 LD_50_)	1:2	80	6/6
20 (~2 LD_50_)	1:1	40	5/6-4/6
20 (~2 LD_50_)	1/0.5	20	4/6-3/6

In summary, properly folded, soluble and active rAahII toxin was produced in a wheat germ cell-free expression system. To be active as a toxin, rAahII toxin has to form four disulphide bonds. The rAahII toxin was only soluble expressed in a construct with an N-terminal GST tag. So far, there were no data available on the successful soluble expression of rAahII toxin in *E.* coli or with a cell-free expression system. The final yield of un-cleaved GST-tagged recombinant protein is ~600 μg in 2 ml PBS buffer solution. After cleavage, we removed the protease, the GST-tag and the un-cleaved protein by ultrafiltration through a membrane with a 10-kDa molecular mass cut-off. The 2 ml flow through was concentrated to 20 μl, which finally yielded only 4 μg of purified cleaved rAahII toxin. For future experiments, alternative membranes need to be tested for concentrating the protein. Further up-scaling with an automatized instrument might also improve the yield by replacing the consumed mRNA and WGE [24]. Nevertheless, with the expression of rAahII toxin in a wheat germ cell-free extract, we have been producing an important toxic protein with an extremely low endotoxin level, which is certainly an advantage for follow-up animal testing, and for immunotherapy investigations. In general, the wheat germ *in vitro* translation method that we described is simple and effective, and might form an excellent opportunity to obtain soluble and active recombinant toxic proteins that has to form multiple disulphide bonds.
